# Molecular Mechanisms of Zinc Oxide Nanoparticle-Induced Genotoxicity Short Running Title: Genotoxicity of ZnO NPs

**DOI:** 10.3390/ma10121427

**Published:** 2017-12-14

**Authors:** Agmal Scherzad, Till Meyer, Norbert Kleinsasser, Stephan Hackenberg

**Affiliations:** Department of Oto-Rhino-Laryngology, Plastic, Aesthetic and Reconstructive Head and Neck Surgery, University of Wuerzburg, 97080 Wuerzburg, Germany; scherzad_a@ukw.de (A.S.); meyer_t2@ukw.de (T.M.); kleinsasser_n@ukw.de (N.K.)

**Keywords:** zinc oxide nanoparticles, genotoxicity, DNA damage, ROS, autophagy

## Abstract

Background: Zinc oxide nanoparticles (ZnO NPs) are among the most frequently applied nanomaterials in consumer products. Evidence exists regarding the cytotoxic effects of ZnO NPs in mammalian cells; however, knowledge about the potential genotoxicity of ZnO NPs is rare, and results presented in the current literature are inconsistent. Objectives: The aim of this review is to summarize the existing data regarding the DNA damage that ZnO NPs induce, and focus on the possible molecular mechanisms underlying genotoxic events. Methods: Electronic literature databases were systematically searched for studies that report on the genotoxicity of ZnO NPs. Results: Several methods and different endpoints demonstrate the genotoxic potential of ZnO NPs. Most publications describe in vitro assessments of the oxidative DNA damage triggered by dissoluted Zn^2+^ ions. Most genotoxicological investigations of ZnO NPs address acute exposure situations. Conclusion: Existing evidence indicates that ZnO NPs possibly have the potential to damage DNA. However, there is a lack of long-term exposure experiments that clarify the intracellular bioaccumulation of ZnO NPs and the possible mechanisms of DNA repair and cell survival.

## 1. Introduction

Over the past 15 years, nanotechnology has increasingly gained in importance in industry, biomedicine, and research. According to the current definition of the European Union (EU), nanomaterials are natural, incidental, or manufactured materials that contain particles in an unbound state, either as aggregates or as agglomerates. At least 50% of these particles must exhibit one or more external dimension within the size range of 1–100 nm [1]. Surface properties become more important as a function of the size reduction of a material. Thus, nanoparticles (NPs) have completely different mechanical, optical, electrical, magnetic, and catalytic properties compared with larger particles of the same composition. Hence, the bioactivity of NPs significantly differs from that of their fine-size analogues [2]. Zinc oxide (ZnO) NPs are among the most commonly used nanomaterials in industrial applications. Despite their increasing usage in consumer products, the safety aspects of ZnO NPs remain uncertain. In particular, information regarding the possible genotoxicity of ZnO NPs is rare, and partially contradictory. The aim of this review is to summarize the literature published between 2009 and 2017 that covers the genotoxicity of ZnO NPs in mammalian and non-mammalian in vitro and in vivo systems, and to estimate the current risk of using ZnO NPs in consumer products. Furthermore, information on the molecular mechanisms of ZnO NP-induced DNA damage will also be outlined and discussed.

## 2. Application of ZnO NPs

ZnO forms a whitish powder and has quite a broad spectrum of applications. ZnO formulations are particularly important in the production of rubber, as an additive in cement, and as a main ingredient in pigments and paints. They are also used as catalysts in the chemical industry, and as standard materials in both the pharmaceutical and cosmetic industries. Numerous electronic components contain ZnO due to its favorable semiconductor properties [3]. A further eminent characteristic is its ability to reflect UV irradiation, which makes ZnO an important physical UV filter in sunscreens. Nanoparticulate ZnO has a very high UV-protective value, and is not as occlusive and whitish as bulky ZnO powder. Thus, ZnO NPs are preferentially applied in cosmetic products compared with larger particles. For consumers, skin exposure is the most likely way to come into contact with ZnO NPs, whereas for industrial workers, airway exposure is more relevant [4].

Currently, approximately 1800 industrial products are available that contain nanomaterials [5]. According to article 16 of the Cosmetic Regulation from 2013, cosmetic products containing nanomaterials have to be notified. Prior to 2013, there was no legal requirement for the declaration of NPs in consumer products, and the number could only be estimated. The EU is currently discussing the introduction of such a regulation in order to facilitate the information flow to the public and research institutions. According to consumer product inventories, there are approximately 40 products available on the United States (US) market containing ZnO NPs.

## 3. Exposure Routes

For the toxicological evaluation of NPs, knowledge regarding the routes of intake is essential. Knowledge regarding its bioavailability and resorption is also important. Possible intake routes of NPs in humans are the gastrointestinal tract, the skin, and the airways. For consumers, dermal exposure is the most likely way to come into contact with ZnO NPs due to the high number of cosmetic products containing ZnO NPs. The stratum corneum, known as the upper layer of the skin, seems to be a sufficient barrier against ZnO NP penetration into the epidermis, as shown by several authors [6,7]. It was clearly demonstrated that ZnO NPs were not able to penetrate healthy and intact human or porcine skin. Although NPs may be retained in the hair follicle ostium or skin folds, they are usually sufficiently eliminated by sebum flow [8]. However, skin damages, for example after excessive sun bathing, may harm this protection layer, and lead to possible toxicological effects from NPs. Cytotoxic or genotoxic effects only seem to be relevant in proliferating cells, which can be found in the basal layers of the epidermis. This is why the application of ZnO NPs to injured or defective skin is discussed as being potentially dangerous. The ingestion of ZnO NPs and contact with intestinal mucosa must be evaluated equally. In particular, chronic intestinal illness may lead to a defect in the mucosa barrier, which consequently may lead to an enhanced toxicity. Further studies are needed to evaluate the correlation between the grade of skin damage and the hazard of ZnO NPs.

Airway exposure via inhalation is the predominant means of contact for workers in the chemical, cosmetic, or paint industries [4]. Nanosized particles are able to reach the peripheral airway sites, such as the bronchiolar and alveolar regions. If not carried away by mucociliary transport mechanisms, NPs may affect alveolar cells and cause toxic, genotoxic, or inflammatory effects [4]. Inhalation exposure to ZnO NPs seems to be an important hazard, and risk assessment is urgently needed within this context [9]. Indeed, the airway exposure of NPs seems to be very important in the toxicological circumstances. According to Vermylen et al., the intake of superfine structures via inhalation has profound negative local and systemic side effects, such as an enhanced risk of cardiovascular diseases [10]. These very small particles are able to penetrate the tracheobronchial tree. In particular, ultrafine particles, which have a diameter less than 100 nm, are able to pass directly into the blood stream [10,11]. Some studies hypothesize that NPs might be able to reach the brain along peripheral nerves [12,13]. This may offer a therapeutic option as well. However, toxicological evaluations are warranted.

## 4. Genotoxicity of ZnO NPs

The difference between the volume and surface of NPs enables their variety of chemical, physical, and biological properties [14]. Due to their small size, large surface area, and physicochemical characteristics, NPs may exhibit unpredictable genotoxic properties. The biological properties depend on the manufacturing procedure, agglomeration and aggregation tendencies, and surface coating. During the manufacturing processes, the particle diameters are not homogeneous. Due to their surface, NPs tend to aggregate, which implicates the need for dispersions. Surface coating is a suitable method for preventing the aggregation of NPs. These above-mentioned circumstances significantly influence the toxicity of NPs. Kwon et al. showed that small NPs cross the cellular membranes more easily, which leads to an increased potency of DNA damage. Accumulated NPs might be internalized into the cell mainly during the mitosis process. According to Liu et al., a crucial determinant of toxicity is the solubility of ZnO NPs, which is influenced by various factors, including the pH of the environment in tissues, cells, and organelles [15]. ZnO NPs and other particles such as silver are soluble, and may release ions. Unlike silver, Zn is an important component of several enzymes and transcription factors in the human body. After incorporation, ZnO NPs may dissolve into Zn^2+^ and trigger several signaling pathways and cascades, which might lead to an enhanced influx of calcium, gene upregulation, or the release of pro-inflammatory markers [16]. The solubility of NPs such as silver (Ag), copper (Cu), or ZnO is one of the main contributors to their toxicity. Ag, Cu, and ZnO NPs have some commonalities. Their elemental composition is metallic, they fight the growth of microorganisms, they have a negative surface charge, and most importantly, all of them are soluble [17]. Nevertheless, there are also differences between these metallic particles. According to Bondarenko, although their particle size is similar, their toxicity is likely different. Cu ions may be involved in electron-transfer processes, in contrast to Ag and Zn [17].

According to Golbamaki, the genotoxic effects of NPs may be classified as either “primary genotoxicity” or “secondary genotoxicity”. Reactive oxygen species (ROS) generation during particle-induced inflammation is the cause of secondary genotoxicity, whereas primary genotoxicity refers to genotoxic effects without inflammation [18]. There are studies that point to the correlation between particle size and toxicity [9]. However, information concerning the size dependency of NP-induced toxicity is contradictory. Warheit et al. did not observe any variation in the toxicity levels of large and small TiO2 NPs [19]. However, Golbamaki and Karlsson detected significantly increased DNA damage after cell exposure to larger micrometer-sized particles compared with smaller NPs [18,20]. Due to these inhomogeneous statements, the size dependency of nanotoxicity and nanogenotoxicity needs to be clarified. NP size must always be characterized exactly in order to provide comparable data in the context of the current literature.

Over the past 10 years, studies focusing on the nanotoxicity of ZnO have been continuously published. However, most of these studies primarily address the cytotoxic aspects of ZnO NPs. Dose–response correlations between ZnO NP concentration and cellular viability are investigated in most studies. However, DNA damage occurs at significantly lower concentrations compared with cytotoxic effects. Hence, genotoxicological evaluations of NPs must be performed at non-cytotoxic doses. Although ZnO NPs are frequently applied in industry and research, data on the genotoxic potential of this material is quite limited [21].

### 4.1. Molecular Mechanisms of Genotoxicity and Evaluation of Oxidative DNA Damage

It is crucial to understand the molecular mechanisms of genotoxicity caused by ZnO NPs in order to provide a valid risk assessment. Although several groups have contributed data towards elucidating these pathways, the associated mechanisms and correlations still remain unclear. The role of Zn ions cannot definitely be ruled out at this stage. Auffan et al. showed that chemically stable metallic nanoparticles have no significant cellular toxicity, whereas nanoparticles that are able to be oxidized, reduced, or dissolved are cytotoxic and genotoxic for cellular organisms [22]. Results from the Wuerzburg group suggest a correlation between ion concentration and genotoxic effects [23], but other groups could not confirm these results in several test systems (micronucleus test, comet assay, and γ H2AX) in a human neural cell line [24].

Autophagy is a lysosome-dependent degradation process that is usually activated in stress situations. Roy et al. identified autophagy activation as a major modulator of ZnO NP-induced cellular toxicity [25]. The detection of increased autophagosome formation and several autophagy marker proteins was reported. ROS generation was identified to be a major trigger for the induction of autophagy. Antioxidant enzymes inhibited cell death and reduced autophagy marker protein expression. The important role of autophagy in ZnO NP-induced toxicity was demonstrated by our group as well. Similar to the results reported by Roy et al., cellular damage could be reduced by counteracting oxidative stress and autophagy [26]. The correlation between autophagosome formation and apoptosis is controversially discussed in the literature. According to Vessoni et al., autophagy is a reaction to DNA damage, and plays an ambiguous role in regulating cell fate [27]. On the one hand, autophagy may promote cell protection, e.g., by degrading pro-apoptotic proteins or by supporting DNA repair. On the other hand, autophagy may also lead to cytotoxic events through the degradation of anti-apoptotic and DNA repair-related proteins [28]. In fact, ZnO NP-induced oxidative DNA damage stimulates autophagy pathways, and thus may influence the balance between cell survival and cytotoxicity. Pati et al. demonstrated an inhibition of DNA repair mechanisms. The reduction in the macrophage cell viability was due to the arrest of the cell cycle at the G0/G1 phase, the inhibition of superoxide dismutase, catalase, and eventually ROS [29].

Kononenko et al. demonstrated a concentration-dependent genotoxicity and cytotoxicity. DNA and chromosomal damage was accompanied by a reduction of glutathione S-transferase and catalase activity [30].

The amount of DNA damage does not only depend on the tested NP itself, but also on the exposed target cell, and the cell’s genetic and proteomic properties in particular. ZnO NPs were shown to activate the p53 pathway by several groups [25,31,32]. ZnO NP-induced DNA damage should usually be forced by p53-associated apoptosis. Ng et al. examined a p53 knockdown fibroblast cell line exposed to ZnO NPs, and found a resistance to ZnO NP-mediated apoptosis, as well as a progressive cellular proliferation, indicating a possible first step to carcinogenesis.

The photogenotoxicity of ZnO NPs is a very important topic. UV irradiation was shown to enhance the cytotoxic properties of ZnO NPs in the A549 cell line by Yang and Ma [33]. Wang et al. reported on the oxidative DNA damage induced by ZnO NPs during UVA (ultraviolet) and visible light irradiation in a dose-dependent manner in HaCaT human skin keratinocytes [34]. The authors proclaimed a photogenotoxic potential of ZnO NPs in combination with UV light. These findings must be discussed critically, especially with respect to the use of ZnO NPs in sunscreen products. Contrary results were published by Demir et al., who demonstrated ZnO NP-related DNA damage in human and mouse cell lines using the micronucleus test and comet assay [35]. Furthermore, they observed anchorage-independent cell growth after NP exposure, which can be interpreted as an initial step towards malignant cell transformation. However, UVB exposure antagonized these effects. Future research projects can be expected to illuminate the interactions between UV light and ZnO NPs regarding DNA damage or DNA protection.

Certainly, a detailed characterization of the physicochemical properties of ZnO NPs is crucial in order to understand the partially divergent statements in the literature. Bhattacharya et al. underscored the important role of the physical properties of NPs. They showed that rod-shaped ZnO NPs induced significantly more DNA damage in peripheral blood mononuclear cells compared with spherical NPs [36]. Coatings may also influence the genotoxic potential of ZnO NPs, as shown by Yin et al., who demonstrated the extended DNA damage of NPs coated with poly (methacrylic acid) (PMAA) compared with uncoated particles [37]. The surface activity and large surface area of NPs lead to a high sorption capability, and thus induce further toxic effects. NPs can function as carriers of absorbed toxic substances, and thus enhance their bioavailability [38].

The majority of the current data regarding the genotoxic effects of nanoparticulate ZnO are based on in vitro investigations. In cells, NPs induce inflammation, genotoxic effects, and damages via the generation of reactive oxygen species (ROS). Sharma et al. published several studies on the genotoxicity of ZnO NPs in a variety of cell systems. They observed DNA damage using the single cell microgel electrophoresis (comet) assay in the HepG2 human liver cell line and the A-431 human epidermal cell line. Cells were exposed to ZnO NPs for 6 h [39,40]. The generation of ROS was demonstrated and discussed as a possible trigger of in vitro genotoxicity in both studies. Patel et al. found the generation of ROS in the A-431 cell line following the application of ZnO NPs. In this publication, ZnO NPs induced cell death, as well as a cell cycle arrest in the S and G2/M phase [41]. Tyrosine phosphorylation was shown to be another promoter of DNA damage in HepG2 cells [42]. Transmission and scanning electron microscopy are the usual tools for the investigation of cellular NP uptake, although these methods are quite time-consuming and technically challenging. Condello et al. demonstrated the entrance of ZnO NPs into human colon carcinoma cells, either by passive diffusion, endocytosis, or both. The entrance mode was dependent on the agglomeration state of the nanomaterial [43]. Toduka et al. used side-scattered light in flow cytometry as an indicator of NP uptake into mammalian cells [44]. Several nanomaterials were tested, including ZnO NPs in Chinese hamster ovary (CHO)-K1 cells using this method. Particles were internalized into the cells, and thus induced a high ROS production, which was directly correlated with the genotoxic events shown by the generation of the phosphorylated histone γ-H2AX. The co-cultivation with the antioxidant N-acetylcysteine (NAC) counteracted DNA damage. Kermanizadeh et al. also showed the important role of oxidative stress through demonstrating a suppression of the toxic potential of ZnO NPs by the antioxidant Trolox in a hepatocyte cell line [45]. DNA damage and pro-inflammatory IL-8 production were induced by oxidative stress and ROS generation. Other groups also published similar results demonstrating the positive correlation between oxidative stress and DNA damage [46,47]. The generation of ROS was mainly assessed by the dichloro-dihydro-fluorescein diacetate (DCFH-DA) assay. Various markers for oxidative stress were evaluated, e.g., glutathione (GSH) reduction, elevated gluthatione, malondialdehyde, superoxide dismutase, and catalase. The photogenotoxicity of ZnO NPs, including a high cellular uptake, was shown in Allium cepa [48]. Other groups also demonstrated the connection between DNA damage and ROS production [43,49].

Most of the studies on nanogenotoxicity were performed using cell lines instead of primary cells. Due to high interindividual variation and the difficulty of standardizing cellular harvest, repetitive experiments with large numbers of patients are necessary in order to assess representative data on primary cells. However, primary cells are neither immortalized nor transformed. Thus, the similarity to cells within the origin tissue is usually higher compared with transformed cell lines. This is why studies with primary cells are supplementary to those using standardized cell lines, and can contribute to common knowledge on nanogenotoxicology. Sharma et al. presented a study using primary human epidermal keratinocytes, a relevant target cell for ZnO NPs, which are mainly used in cosmetics applied to the human skin. ZnO NPs were internalized by the cells, as shown by transmission electron microscopy, where they induced a DNA fragmentation after 6 h of exposure at a concentration of 8 µg/mL [50]. Our own group used primary human nasal mucosa to evaluate the genotoxicity of ZnO NPs. Nasal mucosa belongs to the most important primary contact regions of humans with volatile xenobiotics. Cells of the nasal mucosa are representative of the entire human upper aerodigestive tract. Distinct three-dimensional cell culture systems serve to imitate the in vivo situation quite closely [51]. The genotoxic potential of ZnO NPs was proven in human nasal mucosa cells in an air–liquid interface cell culture, as well as by the extended secretion of IL-816. Primary human adipose tissue-derived mesenchymal stem cells showed DNA damage and pro-inflammatory cytokine production after ZnO NP exposure as well. The stem cell migration capacity was impaired significantly after NP exposure. Interestingly, ZnO NPs were internalized into the cells in high amounts after 24 h, and remained in the cytoplasm for over three weeks, indicating bioaccumulation of the particles. Future studies should illuminate cellular uptake dynamics and exclusion mechanisms. The intracellular persistence of NPs could be a severe problem, since even low exposure doses can lead to critical intracellular concentrations after repetitive contact [52]. The repetitive exposure of nasal mucosa mini organ cultures induced an enhanced genotoxic effect, and 24 h after exposure the DNA damage even increased, probably due to persisting NPs in the cells and the ongoing production of ROS [53]. Ghosh et al. investigated the genotoxic effects of ZnO NPs on human peripheral blood mononuclear cells. The in vitro tests revealed weak genotoxic effects. A significant decrease of mitochondrial membrane potential was also detected [54]. Branica et al. demonstrated a significant increase of DNA damage in human lymphocytes after exposure to ZnO NPs [55].

In contrast to the series of publications stating the possible genotoxicity of ZnO NPs, there are other studies showing no evidence of DNA damage. Nam et al. classified ZnO NPs as well as Zn ions as non-genotoxic in the so-called SOS chromotest [56]. In addition, Kwon et al. did not find any genotoxic effects in several in vitro and in vivo assays that used differently sized and differently charged particles [57]. In a study conducted by Alaraby et al., no toxicity or oxidative stress induction was observed in vivo. Furthermore, no significant changes in the frequency of mutant clones or percentage of DNA in tail (comet assay) were measured, although significant changes in Hsp70 and p53 gene expression were detected [58].

Sahu et al. demonstrated the cytotoxic effects and inflammatory potential of ZnO NPs in a human monocyte cell line, but did not observe any DNA damage [59]. Bayat et al. critically discussed the test systems that are routinely used for genotoxicity assessments. They stated that in vitro genotoxicity testing is probably unreliable because different test systems produce inconsistent results [60].

### 4.2. In Vivo Studies

Only a few studies can be found that evaluate the genotoxicity of ZnO NPs in vivo. Pati et al. investigated the toxicity of ZnO NPs in mice. In this publication, ZnO NPs were dispersed in water by vortex mixing. Afterwards, the animals were fed with water containing NPs in order to demonstrate oral exposure. ZnO NP-treated animals showed signs of toxicity, which was associated with severe DNA damage in peripheral blood and bone marrow cells. Moreover, DNA repair mechanisms were inhibited and enhanced organ inflammation was detected, as well as a disturbance of wound healing [29]. Sharma et al. used a mouse model for subacute oral exposure to ZnO NPs for two weeks. NPs accumulated in the liver and induced DNA damage in liver cells. The authors used an Fpg-modified comet assay to prove that oxidative stress induced DNA damage [32]. Ali et al. found a reduction in glutathione, glutathione-S-transferase, and glutathione peroxidase, as well as an increase in malondialdehyde and catalase in Lymnaea luteola freshwater snails after ZnO NP exposure for 24 and 96 h. Genotoxic effects were found in the digestive gland cells treated with ZnO NPs [61]. Li et al. used a mouse model to prove the biodistribution and genotoxicity of orally administered and intraperitoneally injected ZnO NPs [62]. Baky et al. examined the cardiotoxic effects of ZnO NPs in rats [63], and found that alpha-lipoic acid and vitamin E reduced the DNA damage in cardiac cells. Zhao et al. found DNA damage in embryo-larval zebrafish [64]. The authors compared the toxic effects of Zn ions and ZnO NPs, and demonstrated that ions only partially contribute to the toxic effects. In contrast, triethoxycaprylylsilane-coated ZnO NPs did not induce DNA damage in lung cells from rats after inhalation exposure [65]. Ghosh et al. showed a reduced mitochondrial membrane potential in bone marrow cells in vivo. Furthermore, an enhanced oxidative stress, a G0/G1 cell cycle arrest, and chromosomal aberration with micronuclei formation were measured [54]. In the study conducted by Ng et al., a significant toxicity was observed in melanogaster F1 progenies upon ingestion of ZnO NPs. The egg-to-adult viability of the flies was significantly reduced, which was closely associated with ROS induction by ZnO NPs. Nuclear factor E2-related factor 2 was identified to play a role in ZnO NP-mediated ROS production [49]. Anand et al. investigated the effects of ZnO NPs in Drosophila melanogaster. Food containing 0.1 mM to 10 mM of ZnO NPs induced distinctive phenotypic changes, such as a deformed segmented thorax and a single or deformed wing, which were transmitted to offspring in subsequent generations [66]. Manzo et al. investigated the effects of ZnO NPs in sea urchins. ZnO NPs provoked damages to immune cells in adult echinoids and transmissible effects to offspring [67].

## 5. Summary

**Figure 1:**
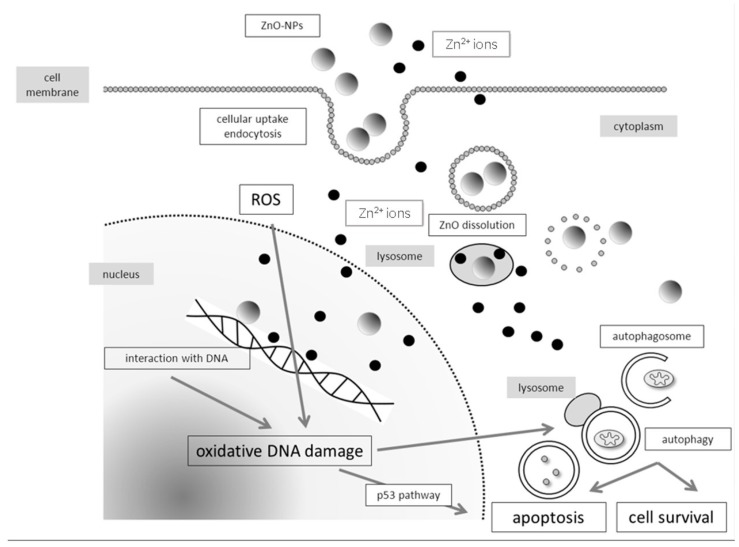
A hypothetical model of Zinc oxide nanoparticle (ZnO NP)-induced genotoxicity.

Although evaluations of the genotoxicity of ZnO NPs are not consistent, there seems to be reliable evidence supporting the potential for them to damage the DNA in human cells. Genotoxic events were demonstrated using several methods and different endpoints. Besides the comet assay, the micronucleus test, the chromosomal aberration assay, and the γ H2AX method were used. The correlation between oxidative stress and DNA damage can be easily proved by the Fpg-modified comet assay and by the interaction with antioxidants such as N-acetylcysteine. Research has shown the internalization of ZnO NPs into the cells via endocytosis or several other mechanisms such as macropinocytosis. Intracellular distribution was observed by transmission electron microscopy as well as by alternative methods such as side scatter flow cytometry. While there is still some controversy surrounding the possible transfer of ZnO NPs into the nucleus, a distribution into cell organelles can definitely be observed. The inclusion into lysosomes seems to be of major importance, since due to the low pH milieu of lysosomes, ZnO dissolves and Zn^2+^ ions are released. Ion release from ZnO NPs may already occur in the cellular expansion medium. Research studies also discuss both intracellular and extracellular Zn^2+^ release as main triggers for DNA damage. Even if ZnO NPs are not able to enter the nucleus, Zn^2+^ ions affect DNA integrity in a dose-dependent manner. Lysosomes release Zn^2+^ ions into the cytoplasm, which is then a trigger for ROS generation. Several research groups have proven this phenomenon by using the DCFH-DA assay. Markers for oxidative stress such as GSH reduction, elevated gluthatione, malondialdehyde, superoxide dismutase, and catalase were analyzed after ZnO NP exposure. As a reaction to disrupted DNA integrity, lysosomes develop into autophagosomes, which can be detected by transmission electron microscopy or indirectly by several protein markers such as LC3 II or beclin-1. The role of autophagy on apoptosis or cell survival is still unclear, and only a few studies address the topic of DNA repair capacity after NP exposure. There is evidence indicating the insufficient repair of DNA disintegrity after ZnO NP exposure, which can be explained by trapped NPs in intracellular departments, and an ongoing trigger for ROS-induced DNA damage. Figure 1 shows a hypothetical model of ZnO NP-induced genotoxicity.

## 6. Conclusions and Recommendations for Future Research

At present, there is still limited information regarding the genotoxic potential of ZnO NPs. Due to inconsistencies in the data available, it is nearly impossible to give recommendations or properly assess the risk of ZnO NP application. Most studies on the hazardous effects of ZnO NPs focus on cytotoxicity. However, ZnO NPs seem to belong to a group of nanomaterials that are able to cause DNA damage. Thus, further genotoxicological evaluation is needed. A strictly detailed and standardized physicochemical characterization of the tested NPs is obligatory in order to produce comparable and informative genotoxicological data. The authors refer to the recommendations of Landsiedel et al. (2010) [65] regarding nanotoxicological study design. Most genotoxicological investigations on ZnO NPs address acute exposure situations. That is why our knowledge of bioaccumulation and long-term exposure effects is only fragmentary. Hence, test systems need to be established in order to clarify these questions, and the biological mechanisms responsible for DNA damage must be analyzed continuously. ZnO NPs are very promising and highly effective materials, and a proper characterization of the genotoxic issues is mandatory in order to apply them reasonably and safely.

Table 1 summarizes relevant publications on ZnO NP-associated genotoxicity mechanisms. The order of listed NPs in Table 1 was sorted according to the particle size, beginning with the smallest. We did not observe any tendency that the results regarding genotoxic potency varied within the two groups of particles smaller or larger than 100 nm. Although the group of larger particles did not exactly fit the definition of NPs, they seem to be still small enough to exhibit comparable toxic properties as compared with NPs <100 nm.

## Figures and Tables

**Table 1:** Current literature review of the genotoxic effects of ZnO nanoparticles.

Characteristics of Nanomaterial(s)	In Vivo	Exposure	Methods	Results	Reference
ZnO NPs: average size 10–20 nm	Earthworm Eisenia fetida (Savigny, 1826)	0.1, 0.5, 1.0, 5.0 g/kg for 7 days	Comet assay	DNA damages were observed at dosages greater than 1.0 g/kg	[66]
ZnO NPs: average size 12 ± 3 nm	Cells of bronchoalveolar lavage fluid, day 1 and 3 after ZnO exposure, in female wild-type C57BL/6JBonTac (C57) mice	Intratracheal instillation of 2, 6, 18 μg ZnO NPs	Comet assay	DNA damage was dose dependent. However, three days post-exposure genotoxicity decreased	[67]
ZnO NPs: average size 22 nm	Freshwater snail *Lymnaea luteola* (*L. luteola*)	10, 21.33, and 32 µg/mL for 96 h	Comet assay	Comet assay revealed DNA damage after treatment with ZnO NPs	[61]
ZnO NPs: average size 28 ± 5 nm Zeta potential −22 mV	Drosophila melanogaster	Food containing 0.1 mM, 1 mM, and 10 mM of ZnO NPs throughout the entire life cycle from egg to egg stage	TUNEL (TdT-mediated dUTP-biotin nick end labeling) assay ROS detection assay	ZnO NPs exposure induced a increase of DNA fragmentation and phenotypic changes, which were transmitted to the offspring	[68]
ZnO NPs: average size 30 nm	Cells of liver and kidney of mice after oral exposure	50 and 300 mg/kg of ZnO for 14 days	Comet assay	The Comet assay revealed a significant increase in the Fpg-specific DNA lesions in liver and kidney cells	[69]
ZnO-NPs: average size: ~70 nm Zeta potential +5.8 mV	MRC5 human lung fibroblasts, Drosophila melanogaster	0, 1, 10, 25, 50, 75, and 100 μg/mL for 24, 48 and 72 h	Comet assay ROS detection assay	Significant genotoxicity was induced by ZnO NPs	[49]
ZnO NPs: average size <100 nm	Human peripheral blood mononuclear cells (PBMCs) and Swiss albino male mice	Cell treatment: 0, 25, 50, and 100 μg/mL for 3 h Animal treatment: 25, 50, and 100 mg/kg body weight 18 h before sacrifice	Comet assay Chromosome aberration assay Micronucleus assay	Apoptosis mediated by ROS generation, reduced mitochondrial membrane potential (MMP) in bone marrow cells, a G0/G1 cell cycle arrest, and chromosomal aberration with micronuclei formation	[54]
ZnO NPs: average size 100 nm Surface area: 15–25 m^2^/g 14 nm Surface area: 30 ± 5 m^2^/g	Sea urchin	1 mg/kg food for three weeks	Comet assay	ZnO NPs 100 nm provoked in adult echinoids damages to immune cells and transmissible effects to offspring, ZnO NPs 14 nm provoked nucleus damages in immune cells and malformed larvae	[70]
ZnO NPs: average size 72 ± 46 nm Zeta potential −13.3 ± 2.3 mV ZnO microparticles particles (MPs)	Madin–Darby canine kidney (MDCK) cells	1, 5, 10, 15, 30, and 60 μg/mL ZnO for 24 h	Comet assay Cytokinesis-block micronucleus assay	ZnO NPs significantly elevated DNA and chromosomal damage, whereas equimolar concentrations of ZnO MPs did not	[30]
ZnO NPs: average size <100 nm	Broodstock zebrafish larvae, Danio rerio	0.2, 1, 2, 4, 6 mg/L for 96 h	Comet assay	Comet assay revealed significant DNA damage after ZnO NPs exposure	[71]
ZnO NPs: average size 20 nm (+) charge: 35 ± 5, 20 nm (−) charge: 28 ± 8, 70 nm (+) charge: 70 ± 19, 70 nm (−) charge: 72 ± 11 nm; Hydrodynamic size of ZnO nanoparticles: 20 nm (+) charge: 200 to 400 nm, 20 nm (−) charge: 180–300, 70 nm (+) charge: 300–900 nm, 70 nm (−) charge: 200–500 nm; zeta potential: 20 nm (+) charge: +25.9 mV, 20 nm (−) charge: −38.5 mV, 70 nm (+) charge: +25.9 mV, 70 nm (−) charge: −40.6 mV	SD rat: liver and stomach cells	500, 1000, and 2000 mg/kg body weights, three times by gavage at 0, 24, and 45 h	Bacterial mutagenicity assay in vitro chromosomal aberration test in vivo comet assay in vivo micronucleus test	Surface modified ZnO NPs did not induce genotoxicity in vitro and in vivo	[57]
ZnO NPs: average size 104.17 ± 66.77 nm	Mouse embryonic fibroblast (MEF Ogg1^+/+^) and mouse embryonic fibroblast knockout (MEF Ogg1^−/−^) cell lines	Sub-toxic dose (1 μg/mL) for 12 weeks, Short-term exposure (0.3125 to 40 μg/mL) for 48 h	Comet assay	Short-term ZnO NPs exposure induce ROS, genotoxicity, and oxidative DNA damage. No effects after long-term exposure	[72]
ZnO NPs: average size 106.55 ± 64.79 nm Zeta potential: −21.00 ± 0.80 mV ZnO NPs bulk: average size 4.2 μm	Haemolymph cells from Drosophila melanogaster	6, 12, 24, mM for 24 h	Wing-spot test Comet assay	No increases in the frequency of mutant spots was detected Significant increase in DNA damage was observed	[73]
ZnO NPs: average size 200–250 nm Zeta potential −0.56 mV	Mice and cells isolated from mice	0–500 µg/mL for 24 h Mice were treated with 200 and 500 mg/kg bodyweight of ZnO NPs	Comet assay Micronucleus Assay	The comet assay revealed severe DNA damage in peripheral blood and bone marrow cells. Moreover, DNA repair mechanism were inhibited	[29]
ZnO NPs: average size 291.66 ± 6.59 nm Zeta potential −11.40 ± 0.26 mV	Drosophila melanogaster	0.02, 0.1, 0.2, 1 and 2 mg/g of food media	The wing-spot assay Comet assay	ZnO NPs were not genotoxic	[58]
ZnO NPs: average size 470 ± 45 nm Zeta potential: −10.35 ± 0.83 mV ZnO NPs: average size 1040 ± 70 nm Zeta potential: −10.51 ± 1.43 mV	Dunaliella tertiolecta	0.1, 2, 5, 10, 25, 50 mg/L for 24 and 72 h	Comet assay	Genotoxic action was evident only starting from 5 mg/L	[74]
ZnO NPs: average size 15–18 nm	Cell line (A549)	0.1, 10, 100 μg/mL	γH2AX immunofluorescence assay	Foci analyses showed the induction of DNA double strand breaks by ZnO NPs. Reduction of DNA damage was achieved by the treatment with the ROS scavenger *N*-acetyl-l-cysteine	[75]
ZnO NPs: average size 15–25 nm	Human neuroblastoma SHSY5Y cell line	20, 30, 40 μg/mL for 3 h and 6 h	Micronuclei evaluation by flow cytometry γH2AX assay Comet assay Oxidative DNA damage	Micronuclei were induced by ZnO NPs, H2AX phosphorylation and DNA damage were observed in all cases	[24]
ZnO NPs: average size 17 nm Zeta potential: −14.0 mV	Human malignant melanoma skin (A375) cell line	5, 10, 20 μg/mL for 24 and 48 h	Comet assay	ZnO NPs induced DNA damage. A gradual nonlinear increase in cell DNA damage was observed as concentration and duration of ZnO nanoparticle exposure increased	[76]
ZnO NPs: average size 10–50 nm	Rat kidney epithelial cell line (NRK-52E)	25.0–100.0 mg/mL for cytotoxicity assays and 12.5–50.0 mg/mL for genotoxicity assay	Comet assay	ZnO NPs caused statistically significant DNA damage	[77]
ZnO NPs: average size 20 nm	Chinese hamster lung fibroblasts (V79 cells)	30.0, 60.0, 120.0 μM for 3 h	Cytokinesis-block micronucleus Assay somatic mutation and Recombination test micronucleus assay	ZnO NPs increase the frequency of micronuclei, results were not dose related	[78]
ZnO NPs: average size 19.6 ± 5.8 nm	Primary mouse embryo fibroblasts (PMEF)	5 and 10 μg/mL for 24 h	Comet assay	ZnO NPs caused statistically significant DNA damage	[79]
ZnO NPs: average size 25.8 ± 8.9 nm Zeta potential: +17.4 mV	Human intestinal carcinoma epithelial cell lines, SW480 and DLD-1 and the normal human intestinal mucosa epithelial cell line, NCM460	Cell exposure concentrations 62.5, 250, and 1000 μM for 12 or 24 h	Oxidative stress measurement Cell cycle analysis	The elevated ROS levels induce significant damage to the DNA of the cells, resulting in cell-cycle arrest and subsequently cell death	[80]
ZnO NPs: average size 25.12 ± 9.2 nm	Cell line from gill tissue of Wallago attu (WAG)	0, 12.5, 25, 50 mg/L for 24 h	Comet assay Micronucleus assay	ZnO NPs induced DNA damage in a dose dependent manner	[81]
ZnO-S ZnO NPs-S: average size 26 ± 9 nm Zeta potential: +19.2 ± 0.3 mV ZnO NPs-M average size 78 ± 25 nm Zeta potential: +20.0 ± 0.6 mV ZnO NPs-L: average size 147 ± 53 nm Zeta potential: +21.1 ± 0.4 mV	Human lymphoblastoid (WIL2-NS) cells	10 mg/L for 24 h	Genotoxicity-cytokinesis-block micronucleus (CBMN) Cytome Assay	Genotoxicity was significantly enhanced in the presence of the medium-sized and large-sized particles	[82]
ZnO NPs: average size 30 nm Zeta potential: −13.4 mV	Human monocytic cell line (THP-1)	0.5, 1, 5, 10, 15, 20 μg/mL for 3 h	Comet assay micronucleus assays	ZnO NPs induced an enhanced DNA damage and micronucleated cells	[83]
ZnO NPs: average size 30 nm Zeta potential: −26 mV	Human epidermal cell line (A431)	0.008–20 μg/mL for 3, 6, 24, 48 h	Comet assay	ZnO NPs induced an enhanced DNA damage	[39]
ZnO NPs: average size 29 ± 10 nm	WIL2-NS human lymphoblastoid cells	10 μg/mL for 24 h	Comet assay	PMAA-coated ZnO had significant genotoxicity compared to uncoated ZnO	[37]
ZnO NPs: average size <35 nm	Human lymphocyte	1.0, 2.5, 5, and 7.5 μg/mL over 2 weeks	Comet assay Comet-FISH	ZnO NPs induced DNA damage	[55]
ZnO NPs: average size ≤35 nm Zeta potential: +46.2 mV ZnO NPs: average size 50–80 nm Zeta potential: −23 mV	Human embryonic kidney (HEK293) and mouse embryonic fibroblast (NIH/3T3) cells	10, 100, 1000 μg/mL for 1 h	Comet assay Micronucleus assay	ZnO NPs induced a significant of DNA damage with and without enzymes. The frequency of micronuclei was enhanced as well	[35]
ZnO NPs: average size ≤35 nm Zeta potential: +46.2 mV ZnO NPs: average size 50–80 nm Zeta potential: −23 mV	Allium cepa root meristem cells	10, 100, 1000 μg/mL for 1 h	Comet assay	ZnO NPs were genotoxic in a dose dependent manner	[84]
ZnO NPs: average size (given by producer) nanosized (30–35 nm) fine (150–300 nm)	human bronchial epithelial BEAS-2B cells	0.5–3.0 μg/cm^2^ for 48 h Comet assay 3 h to 6 h	Comet assay	ZnO NPs exposure induced DNA damage, fine ZnO did not induced DNA damages	[85]
ZnO NPs: average size 40–70 nm	human peripheral lymphocytes, human sperm cells	11.5, 46.2, 69.4, 93.2 μg/mL for 30 min, simultaneous or pre-irradiation with UV light	Comet assay	ZnO NPs are capable of inducing genotoxic effects on human sperm and lymphocytes. The effect is enhanced by UV	[86]
ZnO NPs: average size 50–70 nm	human colon carcinoma cells (LoVo)	Treatment concentration and duration was not unique e.g., cell death assay: 5 μg/cm^2^ ZnO NPs for 2, 4, and 6 h Zn^2+^ ions release: 5 and 10 μg/cm^2^ for 30 min, 1 h, 2 h, 4 h, 6 h, 24 h	DNA damage assessment by 8-oxodG steady-state levels and γ-H2AX histone phosphorylation	ZnO NPs entered LoVo cells. The simultaneous presence of ZnO NPs and Zn^(2+)^ ions in the LoVo cells induced severe DNA damage	[43]
ZnO NPs: average size 75 ± 5 nm	Human lymphocyte cells	0, 125, 500, 1000 μg/mL for 3 h	Comet assay	1000 μg/mL ZnO NPs induced significant genotoxic effects	[87]
ZnO NPs: average size 86 ± 41 nm; mean lateral diameter: 42 ± 21 nm	Primary human nasal mucosa cells	0.01, 0.1, 5, 10, 50 μg/mL for 24 h	Comet assay	ZnO NPs induced DNA damage in a dose dependent manner	[23]
ZnO NPs: average size <100 nm Zeta potential −33.8 ± 10	Saccharomyces cerevisiae cells		GreenScreen assay Comet assay	GreenScreen assay: No genotoxic effects could be measured Comet assay: ZnO NPs were genotoxic	[60]
ZnO NPs: average size <100 nm (given by producer)	lung fibroblast (MRC5) cell line	0, 0.125, 0.25, 0.5, 1, 2, 4, and 8 µg/mL for 24, 48, and 72 h Colony-forming assay: cells were treated for 10 days.	Immunochemical assay DNA methyltransferase activity Quantification of the 5-mC content in genomic DNA	dose-related decrease in global DNA methylation and DNA methyltransferase activity direct correlation between the concentration of NPs, global methylation levels, and expression levels of Dnmt1, 3A, and 3B genes upon exposure	[88]
ZnO NPs: average 20–200 nm, Zeta potential: 26.9 mV	A549 cells	1, 20, 40 μg/cm (=2, 40, 80 μg/mL) for 4 h; fpg-sensitive sites: 20 and 40 μg/cm after 4 h	Comet assay	ZnO NPs induced DNA damage	[89]
ZnO NPs: average size NM-110: 70–100 nm; NM-111: 58–93 nm Zeta potential: NM-110: −11.5 mV; NM-111: −11.4 mV	HK2-cells	Ten concentrations between 0.16 and 80 μg/cm for 4 h	Comet assay	Increase of tail DNA following nanomaterials exposure	[90]
ZnO NPs: average size 45–170 nm Zeta potential: −15.6 ± 2.4 mV	Human colon carcinoma (Caco-2) cells	CBMN assay: 6.4, 12.8, 22.4, 64.0 μg/mL for 6 or 24 h Comet assay: 6.4, 16.0 μg mL^−1^ for 24 h	CBMN assay Comet assay	ZnO NPs induced DNA damage	[91]
ZnO NPs: average size 120 ± 2.6 nm	Root cells of Allium cepa	25, 50, 75, 100 μg/mL for 4 h	Analysis of mitotic index, micronuclei index and chromosomal aberration index	Dose dependent depression of mitotic index, an increase of pyknotic cells, an increase of micronuclei index and chromosomal aberration index	[48]
ZnO NPs: average size NM-110: 20–250/50–350 nm; NM-111: 20–200/10–450 nm	Human hepatoblastoma C3A cells, in vitro	NM concentrations between 0.16 μg cm^−2^ and 80 μg/cm for 4 h	Comet assay	significant increase in percentage tail DNA	[45]
ZnO NPs: average size 64–510 nm Zeta potential: −25.30 mV	human peripheral blood lymphocytes	50–1000 µg/mL for 24 h (cytotoxicity) 25, 50 and 100 µg/mL for 4 h (genotoxicity)	Comet assay	The smaller NPs are more genotoxic, treatment with vitamin C or quercetin significantly reduces the genotoxicity	[92]
	human peripheral blood lymphocytes	0.01–10 mM for 4, 8, 24 h	Comet assay	ZnO NPs induced DNA damage in a dose dependent manner	[93]
ZnO NPs: average size 250–970 nm Zeta potential 20 mV	human bronchial cells (3D model)	30 μL of a 1.06 mg/mL suspension with a dosage of 50 µg/cm^2^ for 24 to 72 h	Comet assay	ZnO NPs were genotoxic in a dose-dependent manner	[94]
ZnO NPs (50 wt %) were purchased From Sigma-Aldrich (St. Louis, MO, USA). No data about particle size	human promyelocytic leukemia (HL-60) cells, and peripheral blood mononuclear cells (PBMC)	0, 0.05, 5, 10, 15, and 20 mg/L for 24 h	Comet assay	ZnO NPs were genotoxic in a dose-dependent manner	[95]
