# Erratum: Scherzad, A., et al. Molecular Mechanisms of Zinc Oxide Nanoparticle-Induced Genotoxicity Short Running Title: Genotoxicity of ZnO NPs. *Materials* 2017, *10*, 1427

**DOI:** 10.3390/ma13235462

**Published:** 2020-11-30

**Authors:** Agmal Scherzad, Till Meyer, Norbert Kleinsasser, Stephan Hackenberg

**Affiliations:** Department of Oto-Rhino-Laryngology, Plastic, Aesthetic and Reconstructive Head and Neck Surgery, University of Wuerzburg, 97080 Wuerzburg, Germany; scherzad_a@ukw.de (A.S.); meyer_t2@ukw.de (T.M.); kleinsasser_n@ukw.de (N.K.)

There is an error in the title. The correct title of the article [1] is Molecular Mechanisms of Zinc Oxide Nanoparticle-Induced Genotoxicity. We apologize for this error and state that the scientific conclusions are unaffected. The original article has been updated.
